# Histamine receptor 2 blockade selectively impacts B and T cells in healthy subjects

**DOI:** 10.1038/s41598-021-88829-w

**Published:** 2021-04-30

**Authors:** Dihia Meghnem, Sharon A. Oldford, Ian D. Haidl, Lisa Barrett, Jean S. Marshall

**Affiliations:** 1 Dalhousie Human Immunology and Inflammation Group, Department of Microbiology and Immunology, Dalhousie University, Sir Charles Tupper Medical Building, Room 7-C2, 5850 College Street, PO Box 15000, Halifax, NS B3H 4R2 Canada; 2 Senescence, Aging, Infection and Immunity Laboratory, Department of Medicine, Dalhousie University, Halifax, NS Canada; 3 Division of Infectious Diseases, Nova Scotia Health Authority, Halifax, NS Canada

**Keywords:** Translational research, Immunology, Medical research

## Abstract

Histamine receptor 2 (H2R) blockade is commonly used in patients with gastric, duodenal ulcers or gastroesophageal reflux disease. Beyond the gastrointestinal tract, H2R is expressed by multiple immune cells, yet little is known about the immunomodulatory effects of such treatment. Clinical reports have associated H2R blockade with leukopenia, neutropenia, and myelosuppression, and has been shown to provide clinical benefit in certain cancer settings. To systematically assess effects of H2R blockade on key immune parameters, a single-center, single-arm clinical study was conducted in 29 healthy subjects. Subjects received daily high dose ranitidine for 6 weeks. Peripheral blood immunophenotyping and mediator analysis were performed at baseline, 3 and 6 weeks into treatment, and 12 weeks after treatment cessation. Ranitidine was well-tolerated, and no drug related adverse events were observed. Ranitidine had no effect on number of neutrophils, basophils or eosinophils. However, ranitidine decreased numbers of B cells and IL-2Rα (CD25) expressing T cells that remained lower even after treatment cessation. Reduced serum levels of IL-2 were also observed and remained low after treatment. These observations highlight a previously unrecognised immunomodulatory sustained impact of H2R blockade. Therefore, the immune impacts of H2R blockade may require greater consideration in the context of vaccination and immunotherapy.

## Introduction

Histamine receptor 2 (H2R) antagonists are one of the most frequently used medications. They are administered long term for the treatment of gastric ulcer disease, gastroesophageal reflux disease (GERD), non-ulcer dyspepsia and related conditions^1^. H2R antagonists decrease the volume and concentration of gastric acid produced by gastric parietal cells^2^. However, a wide variety of other cell types also express H2R including several immune cells^3^ such as neutrophils^4^, eosinophils^5^, monocytes^6^, dendritic cells^7,8^, NK cells^9^, T cells and B cells^10^. Despite the clinical importance of H2R blockade, the effects of H2R blockade on human immunity are not well defined. Several small clinical reports have suggested that ranitidine treatment can be associated with leukopenia, thrombocytopenia^11^, granulocytopenia^12^ and neutropenia^12,13^. It was also linked to a myelosuppressive effect in 5% of bone marrow transplant patients^14^ and shown to affect hematopoiesis via inhibition of bone marrow GM-CSF production^15^. In human NK cells, in vitro histamine treatment was also shown to enhance cytotoxicity via an H2R-dependent mechanism^9^.

Activation of leukocyte populations through H2R can impact immune responses. Indeed, mouse studies have shown that signaling through H2R plays an important role in shaping the immune response. In monocytes, H2R regulates the induction of inflammatory cytokines after LPS stimulation^16^. It also regulates dendritic cell and macrophage CXCL10 and CCL17 chemokine production^18,19^. H2R is also expressed on CD4^+^ T lymphocytes, most strongly in Th2 cells, where it negatively regulates the production of cytokines associated with allergic disease such as IL-13 and IL-4^17^. Histamine was also shown to have a negative feed-back regulation through H2R in rat mast cells^18^. These effects would be predicted to be inhibited by H2R blockade^19^. At a physiological level, H2R is involved in the regulation and self-renewal of hematopoietic stem cells^20^. Preclinical studies of breast cancer have demonstrated that oral treatment of mice with ranitidine inhibits both primary tumor development and metastasis, in three distinct murine breast tumor models^21–23^. This was associated with a reduction in myeloid derived suppressor cell (MDSC) populations following ranitidine treatment of mice. Furthermore, H2R-blockade was shown to inhibit the histamine-dependent increase in cyclooxygenase-2 in colorectal cancer cells^24^.

Clinically, a therapeutic role for H2R blockade has been indicated in advanced multiple myeloma patients. CD19 is expressed at low levels on these tumour cells and CD19 positive myeloma propagating cells have been implicated as having a key role in progression of this disease^25,26^. Ranitidine alone, or in combination with IL-2, was also shown to reduce tumor progression in advanced malignant melanoma patients^27^. Ranitidine treatment has also been shown to increase survival in head and neck squamous carcinoma^28^. Cimetidine, a less selective H2R blocker, was demonstrated to increase cellular and humoral immune responses during vaccination^29,30^. Recently famotidine, another H2R antagonist, was suggested to have beneficial effects in increasing survival following COVID-19 infections^31,32^. These impacts of H2R blockade could all be mediated through impacts on immune cell populations or function. Despite the widespread use of these drugs, their effects on normal human immune cell populations in vivo are still largely unexplored. We therefore determined the effect of ranitidine treatment on peripheral blood leukocyte populations and soluble immune mediators in healthy volunteers to better understand the immunomodulatory impacts of such H2R blockade.

## Methods

### Study population

Eligible participants were 20–50 years of age, with no medical requirement for H2R antagonist use and no use of ranitidine for greater than 1 week within 6 months of the study start date. Individuals were excluded from the study if they had a current or past diagnosis of porphyria, cancer, immune deficiency disorder, active infection at the time of screening, and known liver, hematologic, or renal diseases. Additional exclusion criteria included history of allergic reaction to any drug including ranitidine or to any ingredient in the formulation, weight in excess of 109 kg, pregnancy, or planned pregnancy or breastfeeding during the study period. Participants had no diagnosed clinically significant diseases or evidence of clinically significant findings on physical examination and/or clinical laboratory evaluations (hematology, biochemistry, electrocardiogram, urinalysis). Subjects with clinical laboratory values not within normal range were only included if such values were without clinical significance as defined by the clinical coinvestigators. All participants were required to have an estimated glomerular filtration rate > 90 mL/min/1.73m^2^. Female volunteers of childbearing potential agreed to self-reported use of contraceptive regimens.

### Study design

A single-center, single-arm, prospective phase 4 study was conducted at the Canadian Center for Vaccinology, Halifax, Canada between 01/05/2018 and 01/12/2019 (ClinicalTrials.gov ID: NCT03145012). The study was submitted on the 26/04/2017 and first posted on the 09/05/2017. Healthy volunteers were given daily weight-based oral ranitidine (Zantac, Sanofi Consumer Health Inc., Laval, Canada) at a maximum of 900 mg/day for 6 weeks. Study visits were at weeks 0, 3 and 6 on treatment, as well as 12-week post-treatment. A peripheral venous blood draw was collected at each visit. The study protocol was approved by the Nova Scotia Health Authority and Isaak Walton Killam Health Centre Research Ethics Boards. All study procedures were performed in compliance with the Tri-Council Policy Statement Guidelines, International Council for Harmonization of Technical Requirements for Pharmaceuticals for Human Use Good Clinical Practice Guidelines, and Health Canada Division 5 Food and Drug Regulations. All participants gave written informed consent prior to participation.

### Complete blood counts

Complete blood counts (CBC) with white blood cell differentials were performed on whole blood samples collected in BD Vacutainer EDTA tubes (BD, Franklin Lakes, NJ) using a Sysmex XN-9000 automated hematology system (Sysmex Canada Inc., Mississauga, Canada).

### Immune phenotyping

Immune phenotyping for MDSC and lymphocyte populations was performed on whole blood cells collected in BD Vacutainer Lithium Heparin tubes (BD) by flow cytometry. Antibodies to CD11b, CD14, CD15, CD33, CD66b, HLA-DR, CD3, CD19, CD56, CD4, CD8, CD16, CD25, CD57, CD314 (NKG2D), CD159a (NKG2A), Fixable Viability Stain 780 and Brilliant Stain Buffer were obtained from BD Biosciences (San Jose, CA). Antibody clones are indicated in Supplementary table S1. CD1d tetramers loaded with synthetic glycolipid PBS57 were obtained from the NIH tetramer core facility (Emory University, Atlanta, GA). For each donor, 150 µL of whole blood cells were incubated with fixable viability stain 780 (BD Bioscience) for 10 min on ice then washed in PBS supplemented with 2% FCS. The Fc receptors were blocked with 3% human immunoglobulin (Hizantra, CSL Behring Canada, Ottawa, Canada) for 10 min. Combinations of fluorescently tagged antibodies (Supplementary Table S1) were prepared in brilliant violet buffer and 50 µl were added to each sample for 30 min. After staining, cells were washed twice with PBS supplemented with 2% FCS (Gibco) and the RBC were lysed by adding 5 ml of ammonium chloride for 30–60 s then washed with PBS supplemented with 2% FCS. Cells were fixed with PBS containing 1% paraformaldehyde overnight at 4 °C then washed and resuspended in PBS supplemented with 2% FCS before analysis on BD LSRFortessa™ (BD). The instrument was calibrated with BD Rainbow Calibration Particles (BD) before each acquisition to ensure consistent readings over time. Data were analyzed using FlowJo Version 10 software (BD). Sample gating strategies are depicted in Supplementary Figure S1. Gating was based on fluorescence minus one control. Absolute numbers of immune cell subsets were calculated from automated total white blood cell counts.

### Multiplex immunoassays and ELISA

Soluble mediator levels were assessed in platelet poor plasma isolated from whole blood samples collected in BD Vacutainer EDTA tubes (BD). Plasma samples were assessed for IL-1β, IL-1RA, IL-2, IL-6, IL-7, IL-8, IL-10, IL-15, M-CSF, MMP-9, CCL2, CCL5, CXCL1, IFN-γ, TNF, VEGF-A, IgA, IgE, IgG1, IgG2, IgG4 and IgM via magnetic Luminex performance assays (ThermoFisher, Oakville, Canada) and G-CSF, (R&D Systems, Oakville, Canada). Samples were read using a Bio‐Plex 200 system (Bio-Rad, Mississauga, Canada) and analyzed with Bio‐Plex Manager 6.0 software (Bio-Rad). Plasma levels of B cell activating factor were measured by ELISA (R&D Systems) according to the manufacturer’s recommendations. For both multiplex and ELISA assays a control plasma sample was included on all plates and used for normalization for detectable analytes that displayed > 10% inter-plate variability. For statistical analysis, values below the LOD were assigned the LOD value for the analyte, adjusted for sample dilution.

### Statistical analysis

Data distribution was determined by the D’Agostino & Pearson omnibus k2 normality test. Depending on normality test results, paired statistical analysis was performed using repeated-measures one-way ANOVA or Friedman’s test with Dunnett's or Dunn's multiple comparison post-hoc tests, respectively, using T0 as control. Differences were considered significant at *P* = 0.05. Statistical analyses were performed on raw data using GraphPad Prism software version 8.0 (GraphPad Software Inc., La Jolla, CA).

## Results

### Study population

A total of 34 subjects were screened and 30 healthy volunteers were enrolled. One patient withdrew 2.6 weeks after study start. Data are presented on the remaining 29 healthy volunteers who completed the study. Participants received ranitidine twice daily at a dosage between 7.58 and 8.5 mg/kg/day, a high dose but lower than the one used in patients with Zollinger Ellison syndrome. The median age of the cohort was 31 years (range 23–47 years), all were Caucasian, and 17% were male (Supplementary Table S2). Self-reported ranitidine adherence was on average 97% adherence (range 89–100%). There were no study drug-related safety issues.

### Ranitidine treatment did not affect the overall leukocyte profile or MDSC numbers

Ranitidine use has been associated with neutropenia, anemia, granulocytopenia and thrombocytopenia in various clinical contexts^13,33^. Complete blood counts and differential white blood cell counts were assessed using automated hematology at baseline, during and following ranitidine treatment. Ranitidine treatment did not significantly alter whole blood hematologic counts (Table 1). Minor changes in the number of reticulocytes (RET), mean capsular hemoglobin concentration (MCHC), and Hematocrit (HCT) were observed over the trial but were within normal clinical ranges. Since ranitidine or famotidine treatment has been shown to regulate MDSCs in murine models^21,34^, MDSC subsets were examined in trial subjects by flow cytometry^35^ to identify early stage MDSC (e-MDSC), polymorphonuclear MDSC (PMN-MDSC) and monocytic MDSC (M-MDSC) (Supplementary Figure S1A). The level of MDSCs are normally low in healthy individuals but increase in cancer patients^36^. As expected, low numbers of circulating MDSCs were observed in these healthy subjects, and their overall numbers changed between some sampling times during the study (Table 1). However, ranitidine treatment did not affect the absolute numbers of MDSC subsets at T3, T6 and T18 when compared to T0 (Supplementary figure S2D–F). Analysis of the percentage of the three different populations showed that the percentage of PMN-MDSC were decreased at T18 (Supplementary Figure S2).

**Table 1:** Whole blood hematologic counts^a^ before, during, and after ranitidine treatment.

	Reference range	T0	T3	T6	T18	ANOVA P-value
WBC (× 10^9^/L)	4.50–11.00	6.51 (5.43, 7.34)	6.43 (5.37, 7.24)	6.82 (5.08, 7.86)	7.07 (4.94, 8.23)	0.915
RBC (× 10^12^/L)	4.50–6.50	4.36 (4.14, 4.67)	4.38 (4.17, 4.80)	4.46 (4.18, 4.63)	4.48 (4.22, 4.81)	0.269
HGB (g/L)	120–180	135 (129, 144)	137 (128, 143)	137 (130, 144)	135 (129, 147)	0.582
HCT (L/L)	0.370–0.540	0.40 (0.38, 0.41)	0.40 (0.39, 0.43)	0.40 (0.38, 0.43)	0.41 (0.40, 0.43)**	**0.020**
MCV (fL)	80.0–97.0	90.3 (86.7, 93.8)	91.1 (88.2, 94.3)	90.7 (87.7, 95.6)	90.8 (87.6, 94.5)	0.465
MCH (pg)	28.0–32.0	30.8 (29.5, 31.9)	30.6 (29.5, 31.7)	30.7 (29.6, 31.9)	30.1 (29.2, 31.7)	0.086
MCHC (g/L)	315–350	342 (336, 349)	338 (331, 346)*	337 (329, 345)**	335 (328, 344)**	**0.003**
PLT (× 10^9^/L)	150–350	247 (227, 291)	251 (227, 270)	260 (236, 273)	256 (229, 287)	0.252
RDW (%)	11.5–14.5	12.8 (12.3, 13.2)	12.8 (12.3, 13.2)	12.7 (12.4, 12.9)	12.6 (12.2, 13.2)	0.461
MPV (fL)	9.0–12.5	10.3 (9.9, 11.1)	10.2 (10.0, 11.2)	10.4 (9.9, 11.0)	10.2 (9.8, 11.0)	0.882
RET (× 10^9^/L)	26.1–96.7	58.57 (49.95, 69.34)	57.08 (46.92, 70.44)	63.18 (52.11, 72.31)	56.63 (48.90, 62.25)	**0.006**
NEUT (× 10^9^/L)	2.00–7.50	3.83 (2.94, 4.44)	3.61 (2.97, 4.17)	3.70 (2.99, 4.73)	3.85 (2.60, 4.78)	0.935
LYMPH (× 10^9^/L)	1.50–4.00	1.84 (1.54, 2.19)	1.72 (1.42, 2.11)	1.77 (1.39, 2.39)	1.86 (1.57, 2.18)	0.716
MONO (× 10^9^/L)	0.10–0.90	0.46 (0.40, 0.52)	0.46 (0.38, 0.52)	0.49 (0.44, 0.55)	0.40 (0.35, 0.56)	0.353
EOSI (× 10^9^/L)	0.00–0.50	0.11 (0.09, 0.14)	0.13 (0.09, 0.19)	0.11 (0.07, 0.17)	0.12 (0.09, 0.18)	0.373
BASO (× 10^9^/L)	0.00–0.10	0.03 (0.03, 0.04)	0.04 (0.03, 0.04)	0.03 (0.03, 0.04)	0.04 (0.03, 0.04)	0.079
IG (× 10^9^/L)	0.00–0.09	0.03 (0.02, 0.07)	0.02 (0.02, 0.05)	0.03 (0.02, 0.05)	0.02 (0.01, 0.05)	0.589
e-MDSC (× 10^6^/L)		11.68 (3.98, 46.3)	18.02 (8.96, 37.79)	13.02 (8.17, 53.19)	26.89 (12.81, 53.19)	0.290
PMN-MDSC (× 10^6^/L)		114.5 (56.21, 327)	144.7 (80.4, 293.1)	76.13 (30.05, 146.2	58 (32.28, 153.4)	**0.013**
M-MDSC(× 10^6^/L)		4.86 (3.4, 13.59)	10.62 (4.14, 24.08)	5.77 (1.72, 16.60)	3.63 (1.43, 6.74)	**0.005**

*WBC* white blood cell count, *RBC* red blood cell count, *HGB* hemoglobin, *HCT* hematocrit, *MCV* mean corpuscular volume, *MCH* mean corpuscular hemoglobin, *MCHC* mean corpuscular hemoglobin concentration, *PLT* platelet count, *RDW* RBC distribution width, *MPV* mean platelet volume, *RET* reticulocyte count, *NEUT* neutrophil count, *LYMPH* lymphocyte count, *MONO* monocyte count, *EOSI* eosinophil count, *BASO* basophil count, *IG* immature granulocyte count.

Statistical analysis was performed using a Friedman’s test (indicated p-values), or where data distribution was appropriate using repeated measures, one-way ANOVA followed by Dunnett’s multiple comparison, using T0 as control. *P < 0.05, **P < 0.01 compared to T0.

^a^CBC with WBC differential counts were assessed before ranitidine treatment (T0), after 3- and 6-weeks of treatment (T3 and T6), and after 12 weeks treatment cessation (T18). Data are presented as median (interquartile range).

### Ranitidine treatment was associated with a decrease in B lymphocytes but not immunoglobulin levels

**Figure 1:**
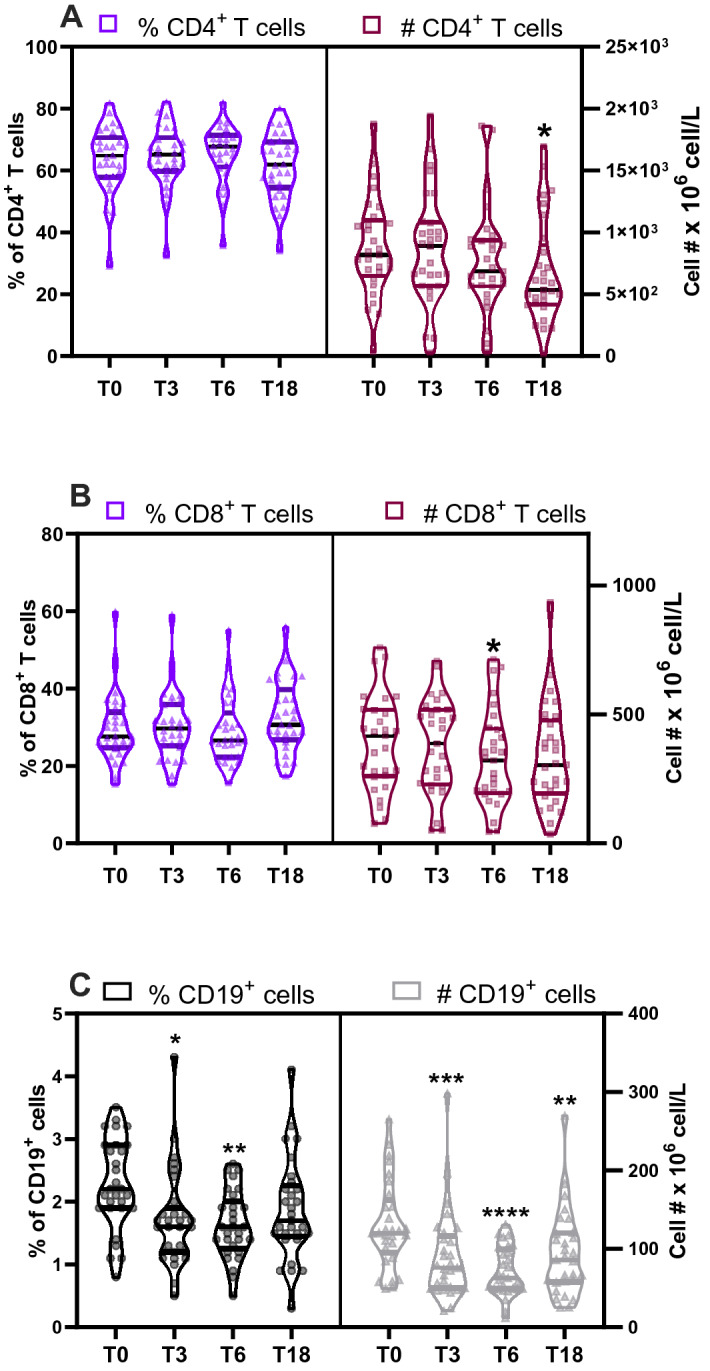
Ranitidine treatment was associated with a substantial decrease of CD19^+^ B cells: The percentage and number of peripheral blood CD4^+^ (**A**), CD8^+^ T cells (**B**) and CD19^+^ cells (**C**) were assessed before ranitidine treatment (T0), after 3- and 6-weeks treatment (T3 and T6), and 12 weeks after treatment cessation (T18) by flow cytometry. Statistical analysis was performed using repeated measures Friedman’s test with Dunn's multiple comparison using T0 as control. Graphs depict median and IQR, n = 29. *P < .05; **P < .01; ***P < .001; ****P < .0001.

H2R is known to regulate B cell activation, antibody production and class switch in experimental models^33^. We examined the effects of ranitidine on B cells and T cells by flow cytometry (Supplementary Figure S1B). Ranitidine treatment decreased the absolute numbers of CD4^+^ and CD8^+^ T cells at T6 and T18, respectively (Fig. 1A,B right panels). However, it did not significantly affect their percentages (Fig. 1A,B left panels). Interestingly, both the numbers and percentage of CD19^+^ B cells were profoundly altered by ranitidine treatment. The absolute number of B cells decreased after 3 and 6 weeks of ranitidine treatment and this decrease was maintained after treatment cessation (Fig. 1C) with a post-treatment T18 trend towards T0 baseline levels. Given the notable decrease in B cells observed following ranitidine treatment serum immunoglobulins were also assessed. No significant changes in immunoglobulin levels were observed within the time frame of this study (Supplementary Figure S3).

### H2R blockade is associated with a modest decrease in NK cells in the blood

**Figure 2:**
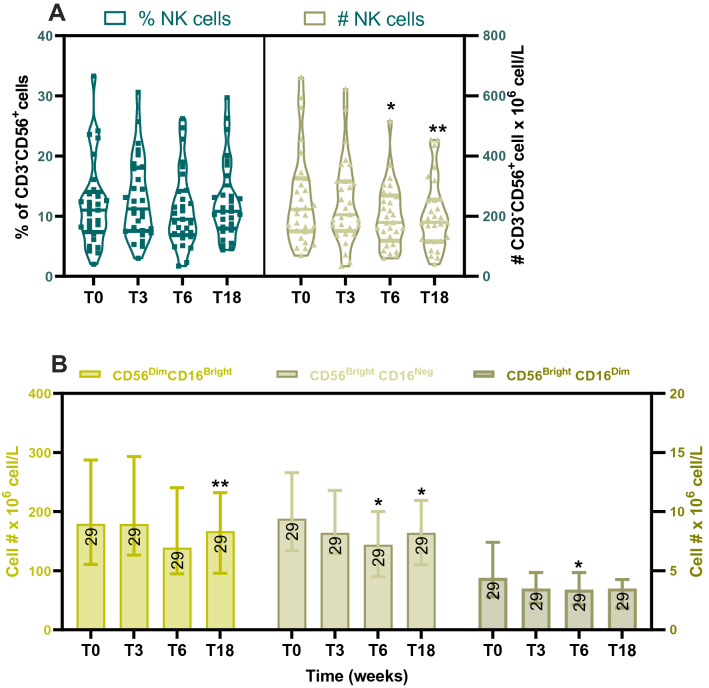
Ranitidine treatment is associated with a decrease of NK cells: The percentage and number of peripheral blood total CD3-CD56 + NK cells (**A**) and their subsets CD56^bright^CD16^neg^, CD56^bright^CD16^dim^ cells and CD56^dim^CD16^bright^ NK cells subsets (**B**) were assessed before ranitidine treatment (T0), after 3- and 6-weeks treatment (T3 and T6), and 12 weeks after treatment cessation (T18) by flow cytometry. Statistical analysis was performed using repeated measures Friedman’s test with Dunn's multiple comparison using T0 as control (A and B) and t-test to compare immunoglobulin level at T0 and T6. Graphs depict median and IQR, n = 29. *P < .05; **P < .01.

Ranitidine treatment did not initially affect the numbers of CD3^-^CD56^+^ NK cells but a modest but statistically significant decrease at T6 and T18 was observed compared to the baseline T0 (T6 *P* = 0.033; T18 *P* = 0.0034, Fig. 2A right panel). CD56^bright^CD16^neg^ NK cells and CD56^bright^CD16^dim^ NK cells are minor NK populations in the peripheral blood and are highly potent cytokine secreting cells^37^. CD56^dim^CD16^bright^ NK cells represent the majority of NK cells in the blood and are highly cytotoxic^38^. Numbers of the predominant NK subsets CD56^Dim^CD16^Bright^ was decreased at T18. CD56^Bright^CD16^neg^ NK cells were decreased at T6 and T18 and CD56^Bright^CD16^Dim^ was reduced at T6 (Fig. 2B). This result indicated no evidence of a selective impact of H2R blockade on a specific NK cell subset.

### ***H2R blockade is associated with altered frequency of CD25 expressing CD4***^+^***and CD8***^+^***T lymphocytes***

**Figure 3:**
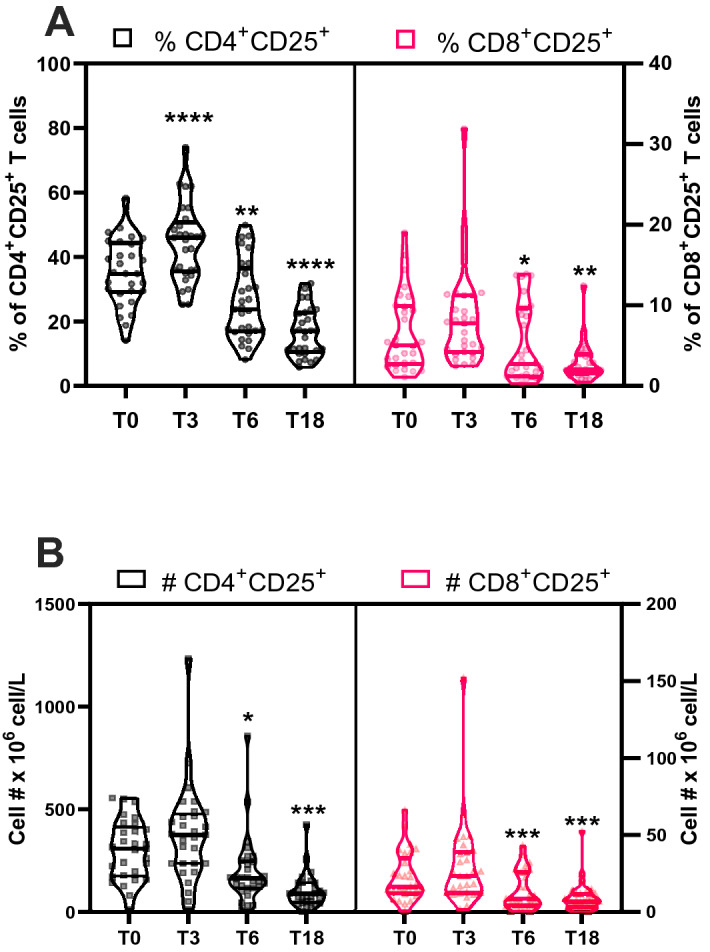
Ranitidine treatment is associated with a substantial decrease in CD25 expressing T cells: The percentage (**A**) and number (**B**) of peripheral blood CD25 expressing CD4^+^ and CD8^+^ T cells were assessed before ranitidine treatment (T0), after 3- and 6-weeks treatment (T3 and T6), and 12 weeks after treatment cessation (T18) by flow cytometry. Statistical analysis was performed using repeated measures Friedman’s test with Dunn's multiple comparison using T0 as control. Graphs depict median and IQR, n = 29. *P < .05; **P < .01; ***P < .001; ****P < .0001.

Ranitidine has been shown to act synergistically with IL-2 to increase NK activity *in vitro*^39^ and increased the efficacy of IL-2 anti-tumor therapy in a clinical study^27^. Therefore, we analyzed the effect of ranitidine treatment on IL-2Rα (CD25) expression in T lymphocytes (Fig. 3). Interestingly, the percentage of CD25^+^CD4^+^ T cells initially increased at T3 before a decline at T6 and T18 (Fig. 3A left panel). Numbers of CD25 expressing CD4^+^ cells were decreased at the end of ranitidine treatment and this decrease was sustained following treatment cessation (Fig. 3B left panel). Numbers of CD25 expressing CD8^+^ cells were also decreased at the end of ranitidine treatment (T6; *P* = 0.0330) and this decrease was sustained at T18 (*P* = 0.0001, Fig. 3B right panel). Similar changes were observed for CD25^+^CD8^+^ percentages (Fig. 3A right panel).

### Selective decrease in serum IL-2 levels associated with ranitidine treatment

Levels of selected soluble immune mediators associated with lymphocyte development and activation or hematopoiesis were assessed in plasma before, during and following ranitidine treatment (Table 2). Levels of immune mediators in subjects following ranitidine treatment were compared to baseline measures in individual subjects. Notably, ranitidine treatment was associated with a selective decrease in plasma IL-2 levels (Table 2). IL-2 was detectable in 25 of the 29 individuals at baseline but was decreased following ranitidine treatment (T3 and T6; *P* < *0.0001)* and remained significantly lower following treatment cessation (T18; *P* = *0.0132*) (Table 2). Interestingly, G-CSF was present at very low levels and detectable above the limit of detection in only 2 of the 29 individuals at T0. G-CSF levels increased following treatment up to 3.61 ± 1.03 pg/ml at T6 (end of treatment timepoint), at which time 31% (n = 9) of subjects had detectable G-CSF levels. At T18 62% (n = 18) of individuals had detectable G-CSF, which was further increased to 6.35 ± 1.02 pg/ml (T18; *P* < 0. 01) compared to T6 or baseline.

**Table 2:** Effects of ranitidine treatment on plasma soluble mediator levels.

	LOD	# above LOD at baseline	T0	T3	T6	T18	ANOVA *P*-value
BAFF	1.43	29	126.7 ± 8.03	117.6 ± 7.47	136.9 ± 11.79	110.5 ± 7.24	0.0562
CCL2	1.69	29	25.15 ± 1.84	23.84 ± 1.82	24.12 ± 2.08	21.12 ± 1.74	0.1582
CCL5	0.19	29	55.9 ± 1.25	53.77 ± 1.90	58.9 ± 1.73	54.07 ± 1.90	0.0109
CXCL1	1.28	0	N.D	N.D	N.D	N.D	–
G-CSF	3.96	2	1.17 ± 0.48	1.09 ± 0.37	3.61 ± 1.03	6.35 ± 1.02**	< 0.0001
IFNγ	6.51	1	7.37 ± 0.86	7.02 ± 0.51	6.51 ± 0.00	6.89 ± 0.38	0.3916
IL-1β	0.86	12	5.47 ± 1.53	3.27 ± 1.09	1.81 ± 0.63	2.05 ± 0.57	0.0239
IL-1R	71.53	0	N.D	N.D	N.D	N.D	–
IL-2	2.45	25	15.24 ± 1.75	8.14 ± 1.46 ****	5.78 ± 0.91****	5.07 ± 1.04*	< 0.0001
IL-6	4.53	1	6.71 ± 2.18	6.002 ± 1.47	5.51 ± 0.89	5.58 ± 1.05	0.3916
IL-7	0.38	28	2.05 ± 0.19	1.68 ± 0.20	1.97 ± 0.18	2.70 ± 0.23	0.0005
IL-8	1.19	0	N.D	N.D	N.D	N.D	–
IL-10	0.73	9	1.04 ± 0.09	0.79 ± 0.04	0.73 ± 0.00	0.73 ± 0.00	< 0.0001
IL-15	1.31	14	8.03 ± 1.90	6.63 ± 1.73	4.98 ± 1.56	4.81 ± 1.60	0.1644
M-CSF	7.14	0	N.D	N.D	N.D	N.D	–
MMP9	0.39	29	58.06 ± 4.21	50.39 ± 4.66	60.35 ± 7.86	54.25 ± 6.78	0.0664
TNF	3.72	4	5.62 ± 1.31	4.61 ± 0.89	3.72 ± 0.56	4.48 ± 0.76	0.0134
VEGF-A	2.34	29	29.33 ± 2.91	31.84 ± 4.24	34.54 ± 5.56	32.01 ± 5.02	0.4599

Plasma soluble mediators were measured before treatment (T0) after 3-and 6-weeks treatment (T3 and T6) and after 12 weeks treatment cessation (T18) by multiplex immunoassay or ELISA. The values depicted represent concentrations (pg/ml) ± SEM. Statistical analysis was performed using a Friedman’s test followed by Dunn’s multiple comparison, or, where appropriate for data distribution, repeated measures one-way ANOVA test followed by Dunnett’s multiple comparison, using T0 as control. *P < 0.05, **P < 0.01, ***P < 0.001, ****P < 0.0001 compared to T0. ND, not detectable (analyte below the LOD in > 85% of individuals, across timepoints).

*LOD* limit of detection.

## Discussion

The effect of ranitidine on human immune cell populations has not been well defined, despite its very common clinical use, especially in the elderly. The present study demonstrates that ranitidine treatment was associated with sustained decreases in CD19^+^ B cells (Fig. 1) and CD25 expressing CD4^+^and CD8^+^ T cells (Fig. 3). However, even at a relatively high dose, ranitidine treatment did not alter total peripheral white blood cell, red cell and platelet parameters (Table 1). The percentage and the absolute number of circulating basophils, neutrophils and monocytes were also not altered by ranitidine (Table 1) although H2R blockade was associated with decreased percentages of PMN-MDSC (Supplementary Figure S1).

To our knowledge, this study defines, for the first-time, the effects of H2R blockade on immune cells in healthy individuals. While ranitidine has been associated with neutropenia in some clinical reports^11,12^, it did not induce neutropenia in healthy individuals over a six week time course (Table 1) even when used at a relatively high clinical dose. Ranitidine has also been associated clinically with thrombocytopenia^40,41^ but we did not observe such impact (Table 1). A sixfold increase in plasma G-CSF levels was observed after cessation of ranitidine treatment (Table 2). While we do not have a clear mechanistic explanation for this G-CSF increase, this change might suggest a compensatory effect for changes in granulocyte populations. We recognise that the dose of ranitidine used in this study was above the one used in a clinical setting and there remain some possibility for off target impacts of ranitidine contributing to our findings. Further clinical immunological studies with alternate H2 antagonists would be necessary to clarify this issue.

This study made the novel observation that ranitidine use was associated with decreased numbers of peripheral CD19^+^ B cells (Fig. 1C). Few studies have analysed the effects of ranitidine on B cells. Ranitidine was shown to modify T lymphocyte function when administered acutely to patients with head injury while having no effect on B cell frequencies^42^. Conversely, in B cell chronic lymphocytic leukemia, ranitidine treatment increased the antibody response to tetanus-toxoid conjugated or unconjugated *Haemophilus influenzae* type-B vaccine^51,52^. Ranitidine also increased antibody responses in a murine tumor model^23^. In these studies, an increase in plasma cells following ranitidine treatment has been suggested. Differentiation of B cells during their developmental stages is supported by IL-7^43^ while their differentiation to plasma cells can be regulated by alternate mediators such as BAFF^44^ and is accompanied by a down regulation of CD19 surface marker^45,46^. The analysis of soluble BAFF and IL-7 in our study (Table 2) did not support an elevation of these mediators in ranitidine treated subjects. Regardless of mechanism, ranitidine impacts on B cells are substantial and might have implications for the humoral immune responses.

H2R blockade has been demonstrated to have some beneficial impacts clinically in the context of multiple myeloma. Although the bulk of multiple myeloma cells do not express CD19 it has been suggested that a critical population of myeloma propagating cells is CD19 positive^47,48^. The notable impact of ranitidine treatment in decreasing CD19 positive cells in the blood could therefore be worthy of further investigation in the context of this disease, where ranitidine treatment has already been suggested to be beneficial^39^.

NK cells are known to express H4R and H2R^49,50^ and their activity can be regulated by histamine receptors antagonists such as ranitidine^50–52^. In this study, NK cells were decreased by ranitidine treatment (Fig. 2A), which was reflected in a decrease in CD56^dim^CD16^bright^, CD56^bright^CD16^neg^ and CD56^bright^CD16^dim^ (Fig. 2B) sub-populations. It was further observed that ranitidine treatment decreased NK cell surface expression of NKG2D (Supplementary Figure S4B). NKG2D is an activating receptor expressed by T cells and NK cells and is important in anti-tumor as well as anti-viral immune responses^53^. The observed effects on NKG2D expression could potentially lead to a decrease in NK and CD8^+^ T cell activation.

MDSCs are induced suppressor cells important in the regulation of immune responses to tumors, inflammation and infections^54^. Strategies targeting MDSCs in cancer have being extensively researched^55^. Although low levels of MDSCs are expected in healthy individuals, our analysis showed that the percentage (Supplementary Figure S2), but not the absolute number of PMN-MDSC was decreased in the peripheral blood after ranitidine treatment (Supplementary figure S2). This finding is similar in nature to the observed impact of ranitidine treatment on MDSC populations in mice^34^ and could contribute to the reported positive impact of ranitidine in some cancer settings.

Ranitidine treatment modulated CD25 expression on T lymphocytes and NK cells (Fig. 3 and Supplementary Figure S4A). A previous study of the postoperative effect of ranitidine on abdominal surgery patients showed an increased plasma level of CD25 in treated patients^56^, indicating a potential role of ranitidine in activation of CD4^+^ T cells and subsequent shedding of CD25. Ranitidine, in combination with IL-2, was also shown to increase NK cell cytotoxicity in vitro^9^ as well as having synergistic effects with anti-tumoral IL-2 therapy^27^. Indeed, frequencies of CD25 expressing cells were increased after 3 weeks of ranitidine treatment (Fig. 3A left panel) which can potentiate IL-2-induced T and NK cell activation. IL-2 itself is a key regulator of CD25 expression and was found to be decreased by three-fold in the plasma after ranitidine treatment. Overall, the effects of ranitidine on B cells as well as CD25 expressing cells and IL-2 plasma levels indicate that H2R blockade profoundly impacts several aspects of acquired immunity.

Ranitidine and other H2R antagonists such as cimetidine and famotidine have previously been shown to affect immune responses to viral infections or vaccination. Cimetidine had beneficial effects during several viral infections^57–59^ and increased immune responses during vaccination^29,30^. Recently, famotidine was associated with beneficial effects in COVID-19^31^ and its use correlated with a two-fold decrease in need for intubation or death^32^. The authors suggested a potential effect of famotidine via decreased cytokine levels^32^. Our results indicate that treatment with the related H2 antagonist ranitidine significantly decreased IL-2 compared to baseline and decreased IL-15 and IL-1β in individuals with higher baseline levels (Table 2). Furthermore, ranitidine has an immunosuppressive effect on NK and T cells demonstrated by a decreased in CD25 and NKG2D expressing cells (Fig. 3 and Supplementary Figure S4). These two phenomena might dampen the immune or inflammatory response to infection and help explain the effect of H2R blockade in COVID-19 infection.

Ranitidine is known to inhibit the constitutive activity of H2R^67^ Such blockade will have a direct effect on H2R expressing cells. Ranitidine can also act on bone marrow cells^14^. For example, hematopoietic stem cells treated with histamine exhibited anti-apoptotic effects on neutrophils and granulocytes^60^. Therefore, inhibiting histamine receptors can lead to altered immune populations in the periphery. The half-life of ranitidine is estimated to be three hours while some observed effects were maintained up to 12 weeks after treatment cessation and these impacts are more likely a result of a long-term effect on the bone marrow microenvironment.

Overall, this study demonstrates that H2R inhibition using ranitidine in healthy individuals induced substantial, prolonged, and multifaceted changes in immune cells to an extent that is likely to be important in immune response regulation. These changes were most notable in the CD19 positive B cell and CD25 positive T cell populations and were associated with decreased plasma IL-2. Our findings indicate that when clinically evaluating immunotherapeutic strategies and immunization responses, greater consideration should be given to histamine receptor antagonists as contributors to variable or modified immune responses.

## Supplementary Information

Supplementary Information
