# Supplementary material for: Histamine receptor 2 blockade selectively impacts B and T cells in healthy subjects
Source: Sci Rep. 2021 Apr 30;11:9405. doi: 10.1038/s41598-021-88829-w (PMC8087813; doi:10.1038/s41598-021-88829-w)
Supplement: Supplementary file 1 — Supplementary Information [file 41598_2021_88829_MOESM1_ESM.docx]

**Title: Histamine receptor 2 blockade selectively impacts B and T cells in healthy subjects**

**Short title:** Immunomodulatory effects of ranitidin**e**

**Authors:** Dihia Meghnem PhD,^1^ Sharon A. Oldford PhD,^2,3^ Ian D. Haidl PhD,^1^ Lisa Barrett MD PhD,^2,3^, Jean S. Marshall PhD

^1^Dalhousie Human Immunology and Inflammation Group, Department of Microbiology and Immunology, Dalhousie University, Halifax, Nova Scotia, Canada; ^2^Senescence, Aging, Infection and Immunity Laboratory, Department of Medicine, Dalhousie University, Halifax, NS, Canada; ^3^Division of Infectious Diseases, Nova Scotia Health Authority, Halifax, Nova Scotia, Canada

**Supplementary table 1: List of antibodies used for immunophenotyping of peripheral blood MDSC and lymphocytes.**

|  | Antibody | Clone | Fluorochrome |
| --- | --- | --- | --- |
| MDSC Panel | CD3 | SK7 | BV785 |
|  | CD11b | ICRF44 | PECY7 |
|  | CD14 | M5E2 | BV605 |
|  | CD15 | HI98 | PECF594 |
|  | CD19 | HIB19 | FITC |
|  | CD33 | WM53 | APC |
|  | CD56 | NCAM16.2 | BV421 |
|  | CD66b | G10F5 | PE |
|  | HLA-DR | G46-6 | AF700 |
|  | Fixable viability dye |  | eF780 |
| Lymphocyte Panel | CD1d tetramer |  | PE |
|  | CD3 | SK7 | BV785 |
|  | CD4 | SK3 | BB700 |
|  | CD8 | RPA-T8 | APC |
|  | CD16 | 3G8 | AF700 |
|  | CD25 | M-A251 | PECF594 |
|  | CD56 | NCAM16.2 | BV650 |
|  | CD57 | NK-1 | BV421 |
|  | CD315 (NKG2D) | 1D11 | AF488 |
|  | Fixable viability dye |  | eF780 |

**Supplementary table 2: Summary of demographic and ranitidine dosage and drug adherence information**

| Study ID | Age (y) | Sex | Body Weight (kg) | Daily Ranitidine dose (mg) | Ranitidine dose (mg/kg/d) |
| --- | --- | --- | --- | --- | --- |
| 1001 | 29 | M | 67.5 | 525 | 7.78 |
| 1002 | 28 | M | 83.8 | 675 | 8.05 |
| 1003 | 34 | F | 107.8 | 825 | 7.65 |
| 1004 | 24 | M | 81.2 | 675 | 8.31 |
| 1005 | 28 | M | 77 | 600 | 7.79 |
| 1006 | 31 | F | 62.2 | 525 | 8.44 |
| 1007 | 23 | F | 85.5 | 675 | 7.89 |
| 1009 | 42 | F | 64.9 | 525 | 8.09 |
| 1010 | 32 | F | 65.4 | 525 | 8.03 |
| 1011 | 37 | F | 73.7 | 600 | 8.14 |
| 1012 | 47 | F | 66.1 | 525 | 7.94 |
| 1014 | 29 | F | 77.7 | 600 | 7.72 |
| 1015 | 28 | F | 78.7 | 600 | 7.62 |
| 1016 | 28 | M | 75.5 | 600 | 7.95 |
| 1018 | 31 | F | 63.3 | 525 | 8.29 |
| 1019 | 46 | F | 62.8 | 525 | 8.36 |
| 1020 | 34 | F | 89.1 | 675 | 7.58 |
| 1021 | 24 | F | 58.7 | 450 | 7.67 |
| 1022 | 27 | F | 64.3 | 525 | 8.16 |
| 1023 | 40 | F | 53.3 | 450 | 8.44 |
| 1025 | 28 | F | 59.1 | 450 | 7.61 |
| 1026 | 31 | F | 68.8 | 525 | 7.63 |
| 1028 | 41 | F | 44.5 | 375 | 8.43 |
| 1029 | 32 | F | 72.2 | 600 | 8.31 |
| 1030 | 34 | F | 37.6 | 300 | 7.98 |
| 1031 | 27 | F | 53 | 450 | 8.49 |
| 1032 | 28 | F | 62 | 525 | 8.47 |
| 1033 | 34 | F | 86.2 | 675 | 7.83 |
| 1034 | 47 | F | 57 | 450 | 7.89 |

**Supplementary figure 1: representative serial gating for the identification of peripheral blood**: **(A)**; MDSC subsets : **Early stage MDSC** are identified as CD11b^+^CD33^+^ among HLA-DR^-^ Lin^-^ (CD3^-^ CD14^-^ CD15^-^ CD19^-^ CD56^-^ cells), **PMN-MDSC** are defined as CD11b^+^ CD66^high^ among CD15^+^ CD14^-^ SSC^low^ cells, **M-MDSC** are defined as CD14^+^ HLA-DR^low/-^ among the monocytic population gated on the basis of FCS and SSC **(B) lymphocytes subsets :** NK defined as CD3^-^CD56^+^ and three subpopulations were defined based on CD16 and CD56 expression CD56^high^CD16^neg^, CD56^high^CD16^dim^ and CD56^dim^CD16^high^. CD8^+^ T cells and CD4^+^ T cell defined as CD3^+^CD8^+^ and CD3^+^CD4^+^ respectively and further analysis on CD3^+^CD4^+^CD25^high^ is illustrated. All gatings were done on live cells and excluded doublets.


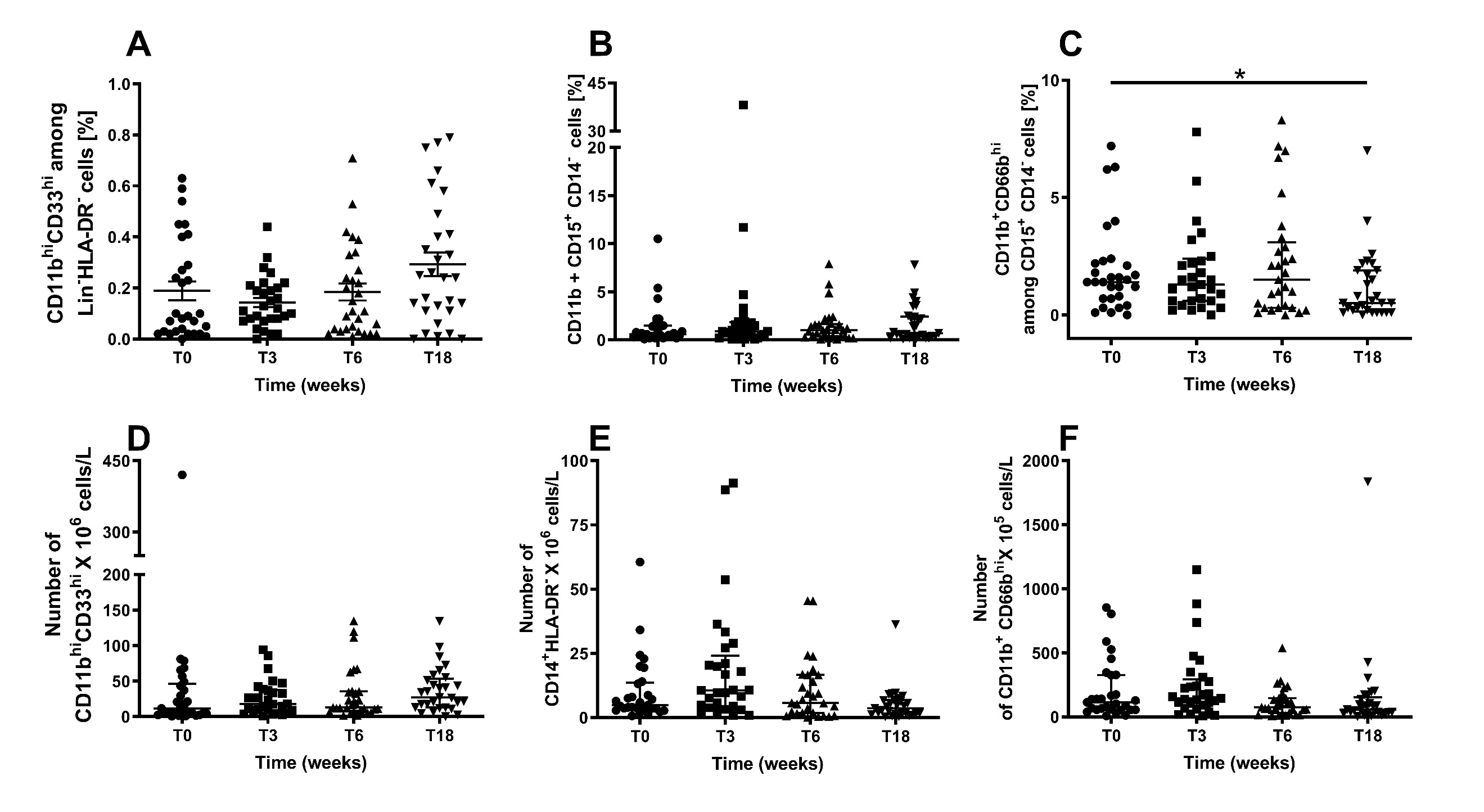


**Supplementary figure 2: Ranitidine treatment reduces the percentage of peripheral blood PMN-MDSC in healthy donors:** Percentage of peripheral blood e-MDSC **(A)**, M-MDSC **(B)** PMN-MDSC **(C)** and their numbers **(D, E, F)** respectively, were assessed before ranitidine treatment (T0), after 3- and 6-weeks treatment (T3 and T6) and after 12 weeks treatment discontinuation (T18) by flow cytometry. Statistical analysis was performed using repeated measures Friedman’s test with Dunn's multiple comparison using T0 as control. Graphs depict median and IQR, n=29. *, P < .05

**Supplementary figure 3: Ranitidine treatment did not affect immunoglobulin levels:** Plasma immunoglobulin levels were measured before treatment (T0) after 3-and 6-weeks treatment (T3 and T6) and after 12 weeks treatment cessation(T18) by multiplex immunoassay. The values depicted represent concentration (pg/ml) and IQR. Statistical analysis was performed using Mann-Whitney test comparison between T0 and T6.


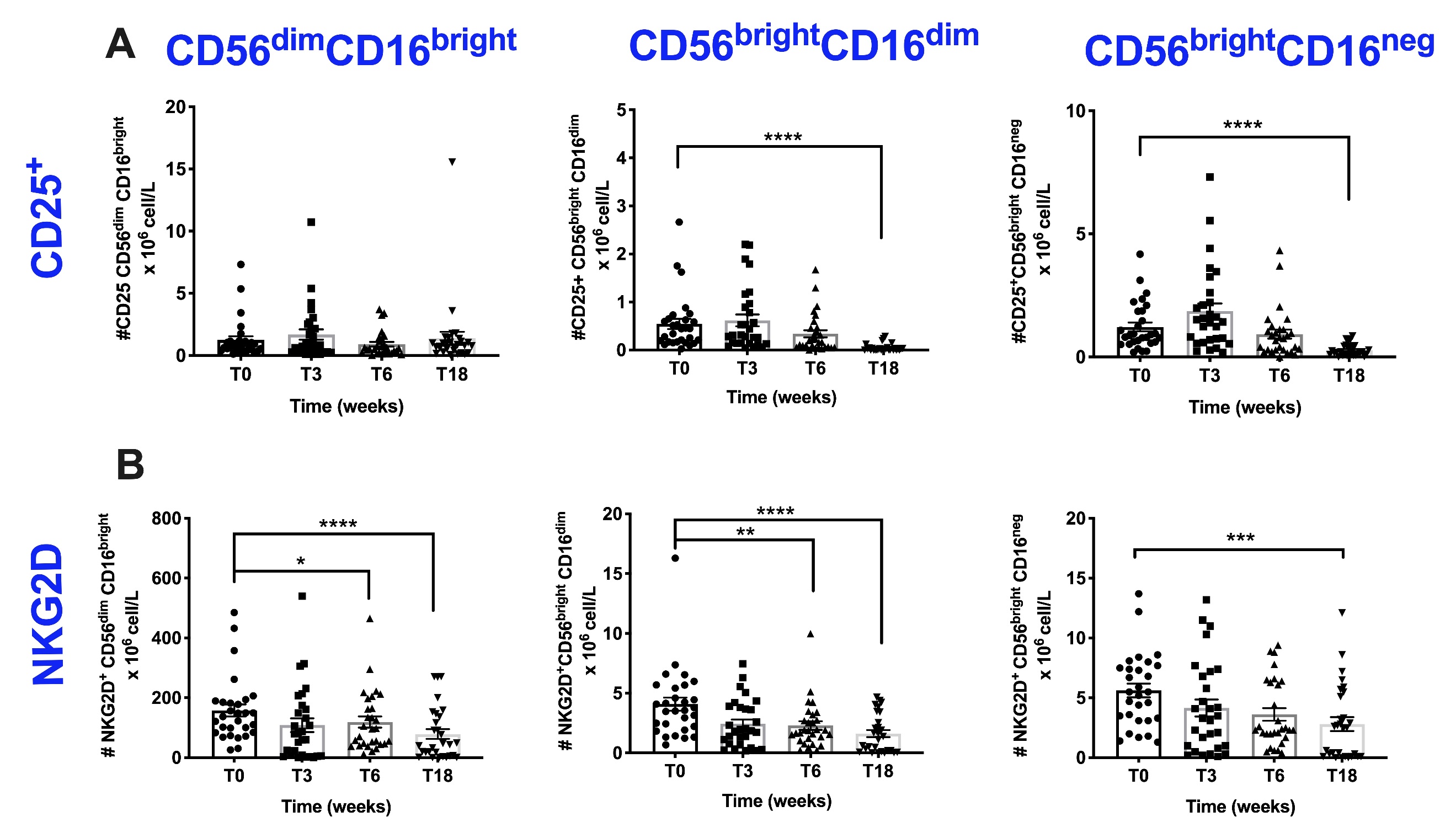


**Supplementary figure 4: Ranitidine treatment decreased NK cell subsets and modulated activation receptors: Expression of** activation markers across NK cell subpopulations are depicted as fellow**:** CD25 **(Panel A)** and NKG2D **(Panel B)** and were assessed before ranitidine treatment (T0), after 3- and 6-weeks treatment (T3 and T6) and 12 weeks after treatment discontinuation (T18) by flow cytometry. Statistical analysis was performed using repeated measures Friedman’s test with Dunn's multiple comparison using T0 as control. Graphs depict means ± SEM, n=29. *, P < .05; **, P< .01***, P< .001 ; ****, P< .0001.

.
